# Supplementary figures and images for: Histone deacetylase 8 interacts with the GTPase SmRho1 in Schistosoma mansoni
Source: PLoS Negl Trop Dis. 2021 Nov 29;15(11):e0009503. doi: 10.1371/journal.pntd.0009503 (PMC8670706; doi:10.1371/journal.pntd.0009503)

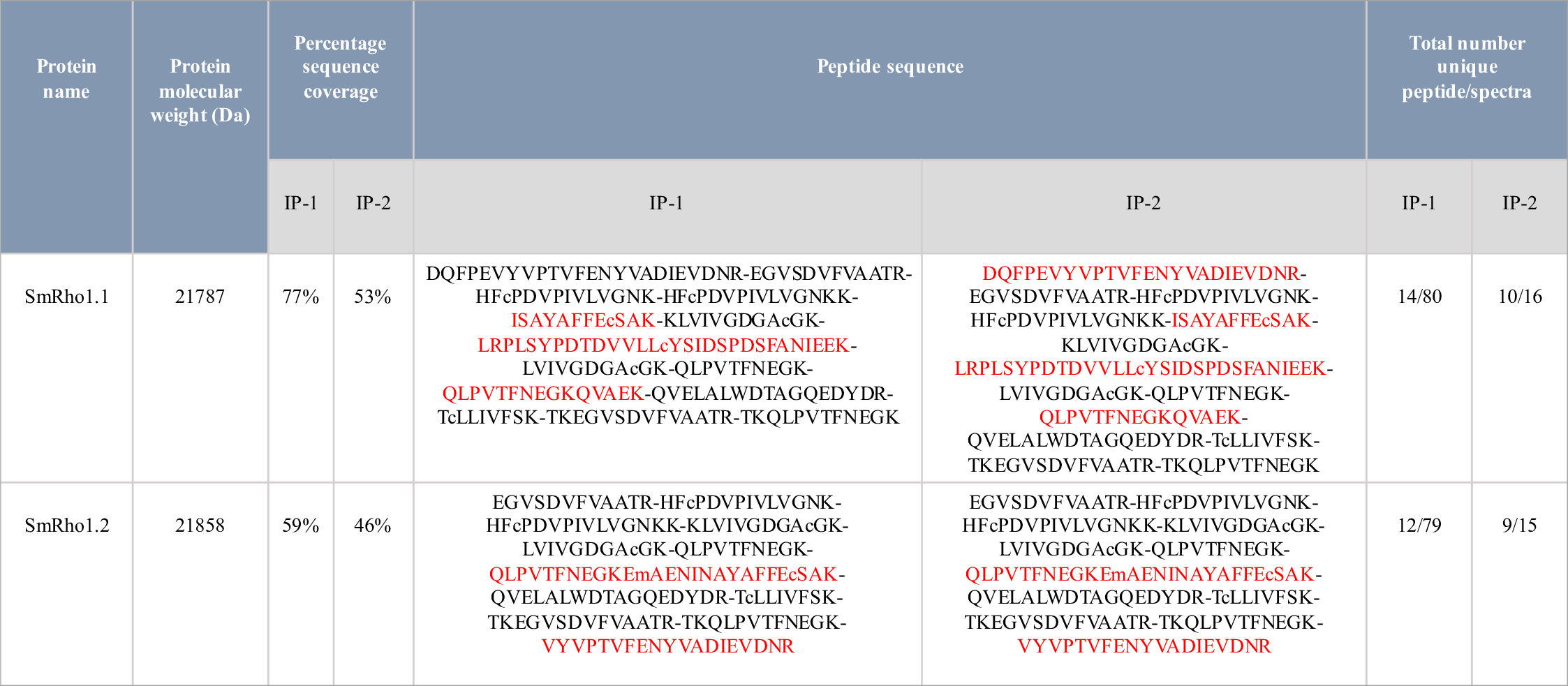

Supplement: S1 Fig — The table indicates the sequence, the molecular weight, the percentage of sequence coverage and the number of “unique peptides/spectra” for each identified protein in two biological independent assays (IP1 and IP2). (TIF) [file pntd.0009503.s001.tif]

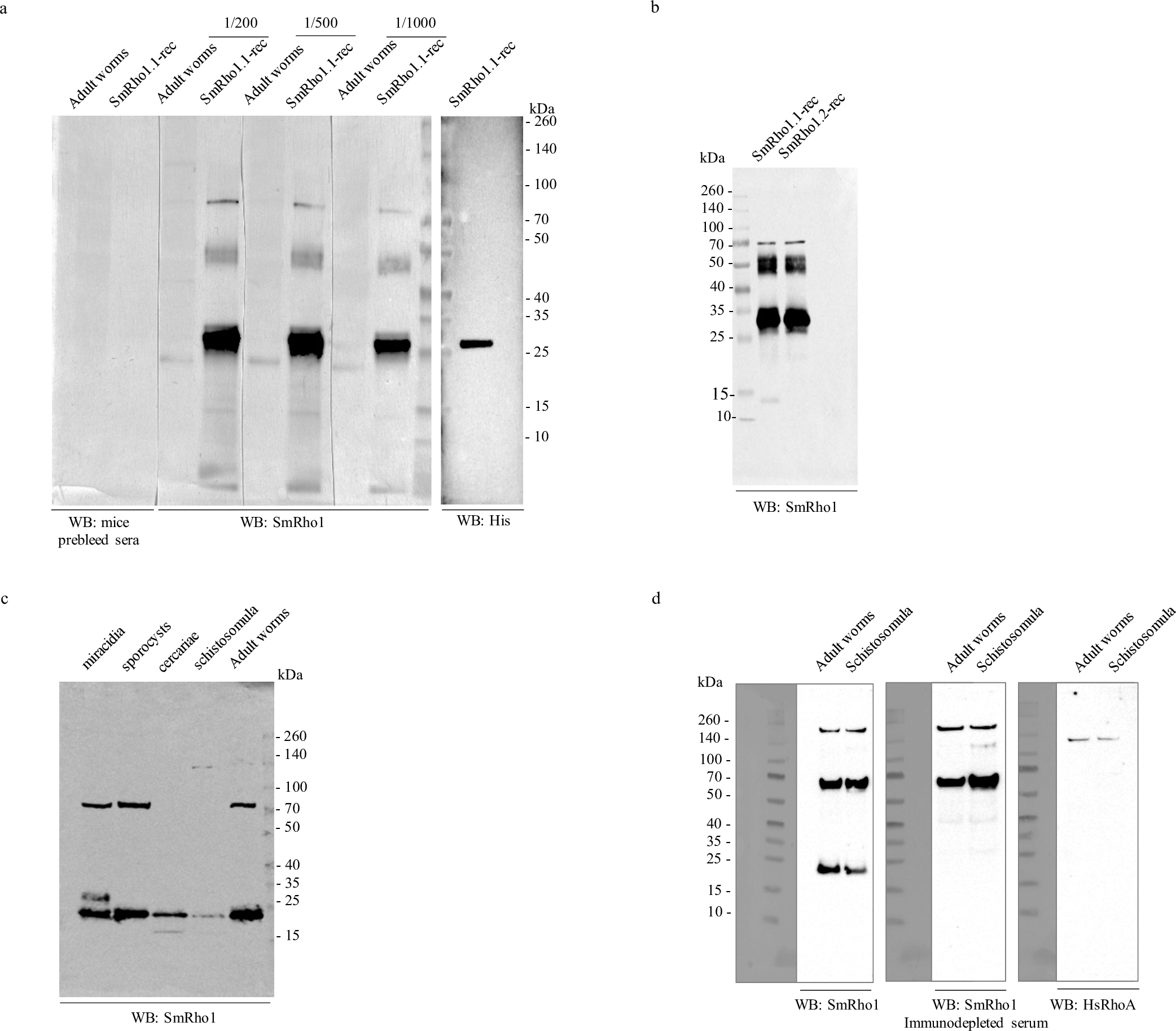

Supplement: S2 Fig — (A) Detection of adult worms endogenous SmRho1 and SmRho1.1 recombinant protein. The blots were probed with mouse prebleed sera, with mouse anti-SmRho1 antisera tested at 1/200, 1/500 and 1/1 000 dilution and with mAb anti-His. (B) Immunoblot analysis of recombinant SmRho1.1 (SmRho1.1-rec) and SmRho1.2 (SmRho1.2-rec) proteins with mouse anti-SmRho1 antisera. (C) detection of endogenous SmRho1 in total proteins extracted from parasitic stages of S. mansoni. (D) Immunodepletion of SmRho1antibodies using SmRho1.1 recombinant protein show the specificity of the antibody against SmRho1. Adult worms and schistosomula endogenous SmRho1 proteins were detected by WB using mouse anti-SmRho1 antisera (left panel) or mouse anti-SmRho1 depleted antisera (middle panel). Human RhoA antibody was used as negative control (right panel). (TIF) [file pntd.0009503.s002.tif]

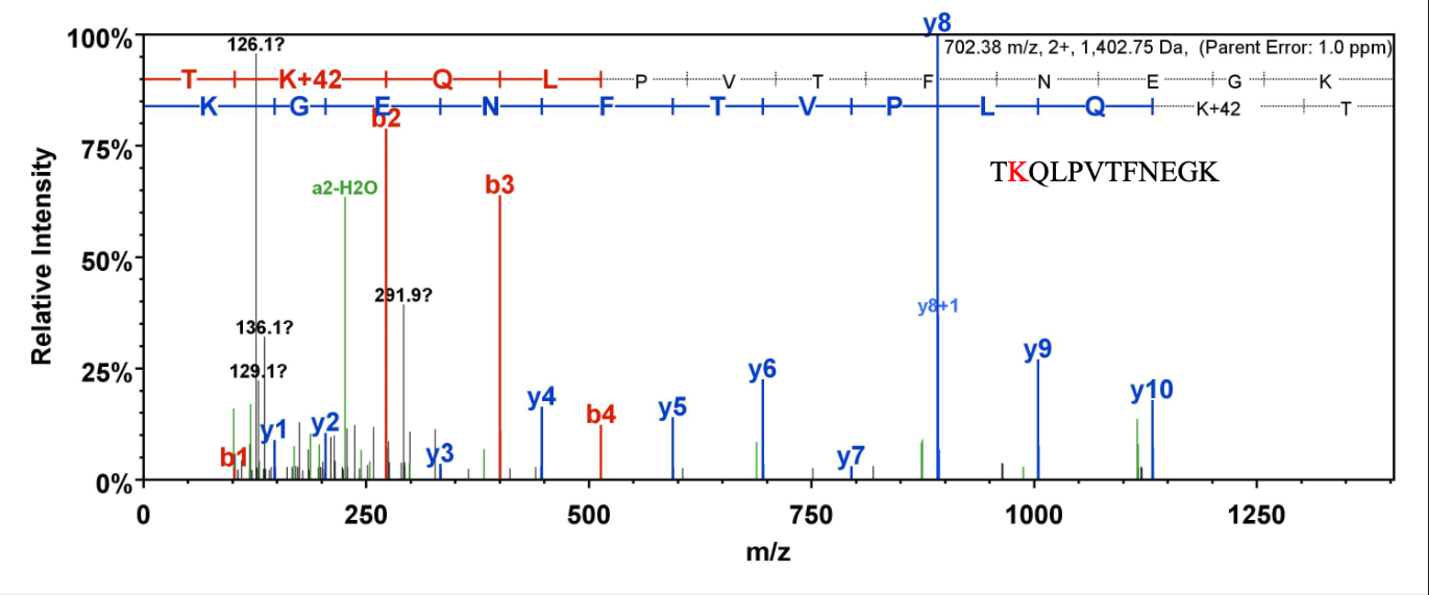

Supplement: S3 Fig — MS/MS spectrum of the acetylated peptide TK(Ac)QLPVTFNEGK. B (red) and y (blue) ion fragments series clearly identified peptide sequence and acetylation on lysine 136 with a Mascot ion score of 60.6. The experiment was performed twice on adult worms obtained by three different hepatic portal perfusions of hamsters. (TIF) [file pntd.0009503.s003.tif]

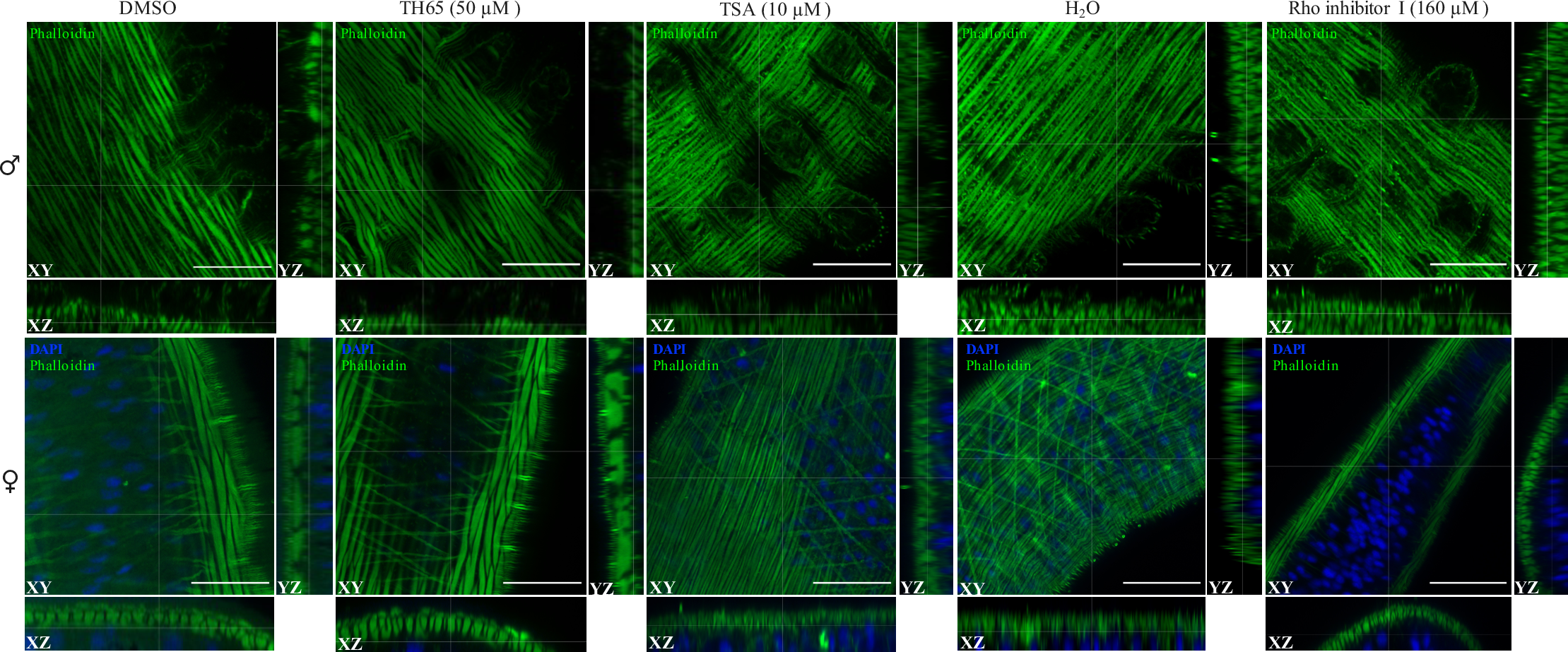

Supplement: S4 Fig — Scale bar represents 20 μm, magnification, x630. Experiments were performed three times on adult worms obtained by three different hepatic portal perfusions of hamsters. (TIF) [file pntd.0009503.s004.tif]

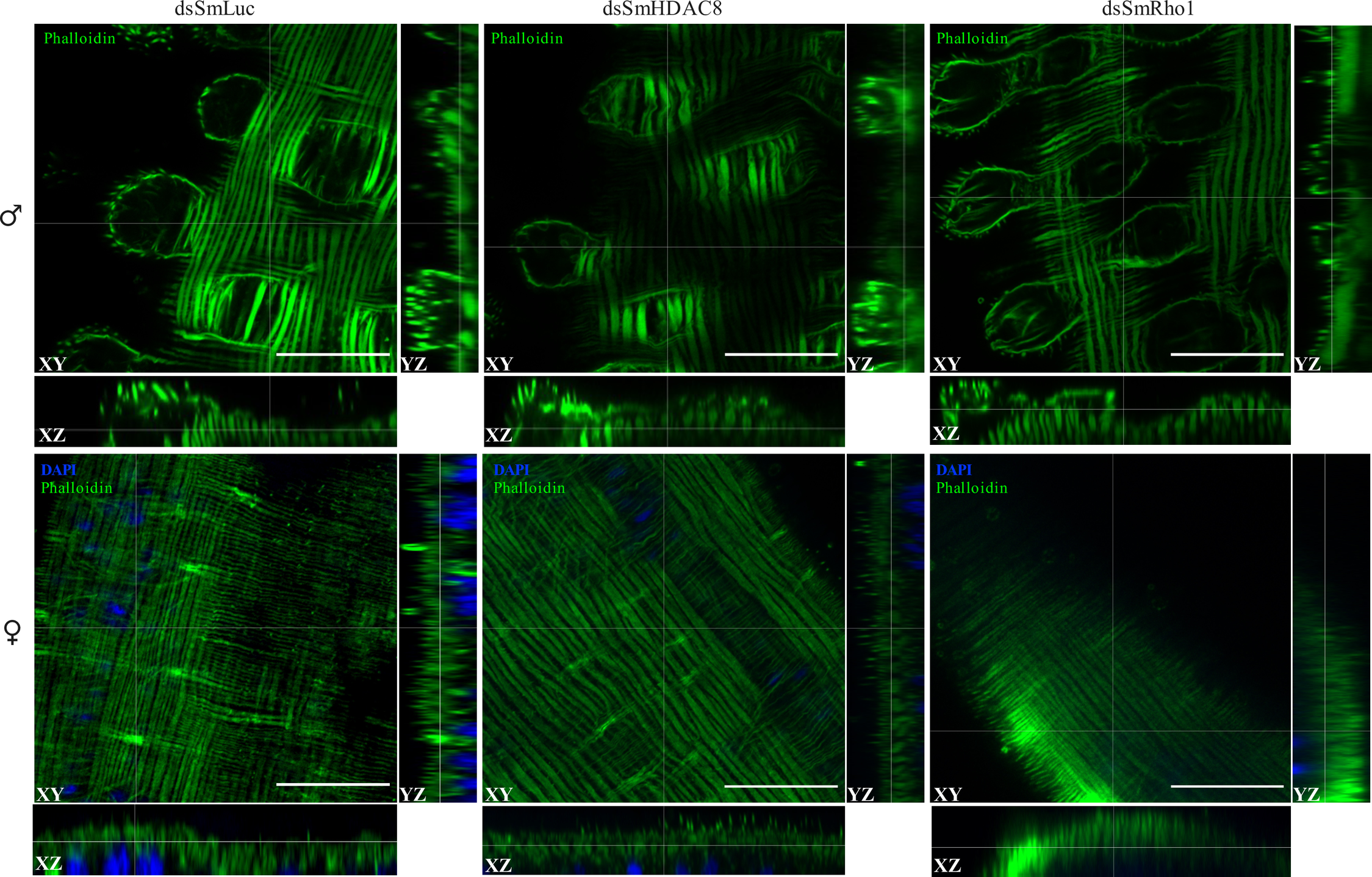

Supplement: S5 Fig — Scale bar represents 20 μm, magnification, x630. Experiments were performed three times on adult worms obtained by three different hepatic portal perfusions of hamsters. (TIF) [file pntd.0009503.s005.tif]

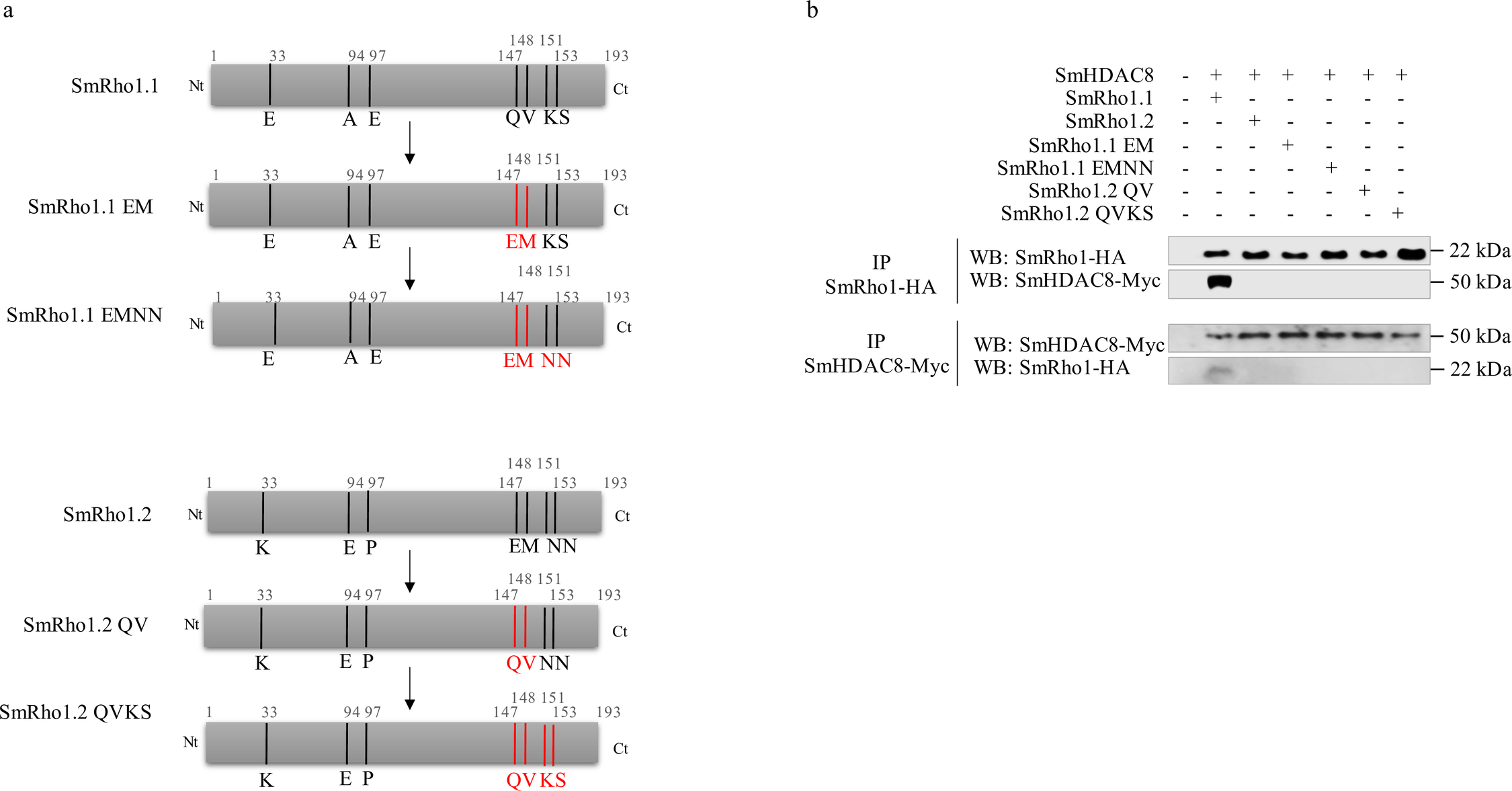

Supplement: S6 Fig — (A) Schematic structure of SmRho1.1 and SmRho1.2 mutants. Using site-directed mutagenesis, the glutamine Q147 and the valine V148 of SmRho1.1 were substituted by a glutamic acid and a methionine (SmRho1.1 EM) and then the lysine K151 and the serine S153 by two asparagines (SmRho1.1 EMNN). SmRho1.2 QV and SmRho1.2 QVKS mutants were produced by site- directed mutagenesis using SmRho1.2 protein. First, the glutamic acid E147 and the methionine M148 were substituted by a glutamine and a valine and then, the two asparagines N151 and N153 were replaced by a lysine and a serine. (B) Co-immunoprecipitation and WB experiments performed in X. laevis oocytes revealed that SmRho1.2 mutants (HA-tagged) are not able to bind SmHDAC8 (Myc-tagged). cRNAs encoding HA-tagged SmRho1 isoforms, SmRho1.1 mutants or SmRho1.2 mutants were co-injected in X. laevis oocytes with cRNA encoding Myc-tagged SmHDAC8. Oocytes were incubated in ND96 medium and lysed. Proteins from soluble extracts were immunoprecipitated (IP) by anti-HA or anti-Myc antibodies and analyzed by WB to detect SmHDAC8 (50 kDa), SmRho1 isoforms (22 kDa) or SmRho1 mutants (22 kDa) with anti-Myc or anti-HA antibodies. (TIF) [file pntd.0009503.s006.tif]
