# Supplementary material for: Histone deacetylase 8 interacts with the GTPase SmRho1 in Schistosoma mansoni
Source: PLoS Negl Trop Dis. 2021 Nov 29;15(11):e0009503. doi: 10.1371/journal.pntd.0009503 (PMC8670706; doi:10.1371/journal.pntd.0009503)
Supplement: Attachment — Submitted filename: Replies to reviewers comments revision 2.docx [file pntd.0009503.s011.docx]

**Replies to reviewers’ comments.**

*Additional remarks:

- Line 738 - "phalloidin staining revealed higher-order actin structures,
forming a three-dimensional actin network." - If we interpreted the
figures correctly, this network seems to be the muscle fibers of the
subtegumental muscle layer - it would seem clearer to describe it as such.*

Done

*- Regarding your reply to the reviewers and the associated changes to the
text:

"Line 411: provide the Smp identifiers for Rho1.1 and 1.2. -> We have
added the Smp identifier for SmRho1.1. For SmRho1.2 no identifier exists
yet."

It would make things clearer to the reader if it was explicitly stated
that SmRho1.2 is not present among the current gene predictions in
Wormbase Parasite, and thus lacks a Smp identifier.*

The text has been changed accordingly

*- Line 752 - "inhibition and KO of SmHDAC8" -> KO should be "silencing" or
"knockdown"*

Changed to “silencing”.

*- Line 819 - "Phosphorylated MLC stimulates the binding of myosin to actin
to regulate actin filament assembly" - MLC phosphorylation typically
regulates stress fibre formation / actomyosin contractility, but not actin
filament assembly. Please revise if necessary.*

Revised as suggested.

*- Line 845 - "...biological processes regulates by a catenin" ->
"...biological processes regulated by a catenin"*

Corrected.

*- Regarding your reply to our previous suggestion:
" Figure 4A - It seems likely that in the left panel, the labels "IP
SmHDAC8" and "IP SmRho1" were incorrectly exchanged ->No, there is no
error"
It seemed strange that a very strong band for HDAC8 and a very weak band
for Rho1 is seen for IP with anti-Rho1, and viceversa, a very strong band
is seen for Rho1 and a very weak band is seen for HDAC8 for IP with
anti-HDAC8.*

The reviewer is correct. We were mistaken in our previous reply and examination of source material shows that the labelling of the lanes for adult worm Co-IP experiments was inverted. We thank the reviewer for insisting on this point and apologize for our obtuseness! The figure has been corrected.

*<b>Summary and General Comments</b></br></br>
Use  this section to provide overall comments, discuss
strengths/weaknesses of the study, novelty, significance, general
execution and scholarship. You may also include additional comments for
the author, including concerns about dual publication, research ethics, or
publication ethics. If requesting major revision, please articulate the
new experiments that are needed.

Reviewer #2: The authors made some efforts to improve their manuscript.
Although not all of my recommendation have been considered (e.g. see
below), this revised version can be accepted for publication.*

*>From my previous review:
Line 606 ff: the authors argue about a parasite protein complex consisting
of SmRho1, SmROCK1, SmLIMK, and the Myosin light chain. If it exists, one
would expect that these partners share the same compartments/organs for
expression. Have the authors searched in the SchistoCyte Atlas
(*[*http://www.collinslab.org/schistocyte/*](http://www.collinslab.org/schistocyte/)*) and/or other sub-transcriptome
resources to find additional support for their assumption? If not, this has
to be done, and the results reported.
Answer by the authors: As suggested by the reviewer we have searched the
SchistoCyte Atlas.
However, we found no relevant data that allow us to link these proteins in
the parasite. We are open to further suggestions, but for this revision we
have not included our analysis.

Looking deeply into the schistocyte data shows interesting patterns of
some of the genes in focus of this study. In some cases, clearly elevated
levels of expression can be seen in cells representing muscle and
parenchyma. In part, expression levels of some of the mentioned genes
appears to be influenced by pairing. Data from this resource would have
supported some of the ideas and conclusions of the authors, and may have
created additional ideas about their biological functions.*

Again, we thank the reviewer for insisting on this point. We have reexamined the schistocyte data and have indeed found that *SmRho1* is coexpressed with *SmROCK1* in parenchyma and muscles and with Smkinesin and Smα-actinin in tegument. Moreover, expression levels seem to be influenced by pairing. We have added this information to the Discussion and have further mentioned the presence of SmRhoGEFs and SmRhoGAPs.

*Reviewer #3: The authors have tremendously improved the manuscript,
changes were implemented (where it made sense), and if not, convincing
reasons were given for not following the suggestions.
The manuscript is much more clear now.

However, there appear still a number of sections of discussion in the
results part. These should be clearly separate from the results and thus
moved to the discussion.
Discussion sections to be moved
- in paragraph from line 786
- whole paragraphs from line 812, from line 817, and from line 821
- lines 899-904
- lines 949-953
- lines 1032-1036*

We have had some difficulty in relating the cited line numbers to the revised version of the manuscript. There may be some confusion since, for instance, lines 1032-1036 are in the Discussion section of the revised manuscript. Nevertheless, we have thoroughly examined the Results section to weed out any remaining text that can be moved to the Discussion. For instance lines 822 to 826 (pdf version of revision 1) concerning the possible distinct functions of the SmRho1 isoforms have been transferred to the Discussion. However, a small amount discursive text has been left in the Results section, notably concerning the summary of the results concerning the interactome (e.g. lines 633 to 639 in the pdf version of the first revision) and the text concerning the identification of RhoGD1 and rab proteins in the interactome. We feel this helps the reader to immediately appreciate the roles and significance of the proteins detected, and is preferable to simply writing out a list of the interactors.

*further minor corrections:
- line 80/81: change to: ... is likely and rendered the development of new
therapeutic agents imperative. (or even better: ... likely, and it calls
for the development of new therapeutic agents)*

We prefer, “rendering the risk of resistance more likely and the development of new therapeutic agents imperative”.

*- line 105: NTD abbreviation not needed, as not further used throughout
manuscript*

Deleted

*- in line 481, and many other places, the dot between weight and volume
needs to be removed still (for example ug mL instead of ug.mL) (also line
548, 563, 564, and further)*

Corrected

*- line 606: state how many technical replicates*

Added to text

*- line 723: delete "and" before human, and replace with a comma*

Corrected

*- line 877: in the Figure it says SmRho1 for both sides of the
venn-diagram. either figure or legend incorrect. please correct.*

Corrected

*- Figure 5 is not central, and should be moved to supplementaries*

Done. Fig. 5 is now Fig. S3.

*- line 934: change to MS/MS*

Again, the line number given does not correspond, but we have verified this throughout the text.
