# Supplementary material for: Histone deacetylase 8 interacts with the GTPase SmRho1 in Schistosoma mansoni
Source: PLoS Negl Trop Dis. 2021 Nov 29;15(11):e0009503. doi: 10.1371/journal.pntd.0009503 (PMC8670706; doi:10.1371/journal.pntd.0009503)
Supplement: Attachment — Submitted filename: Reviewer comments word file_final.docx [file pntd.0009503.s010.docx]

```
Dear Dr. Pierce,

Thank you very much for submitting your manuscript "Histone deacetylase 8 interacts with the GTPase SmRho1 in Schistosoma mansoni" for consideration at PLOS Neglected Tropical Diseases. As with all papers reviewed by the journal, your manuscript was reviewed by members of the editorial board and by several independent reviewers. In light of the reviews (below this email), we would like to invite the resubmission of a significantly-revised version that takes into account the reviewers' comments.

All reviewers were generally positive about the manuscript. However, several major points were raised which should be addressed in a revised version of the manuscript. The question of the specificity of the antibody against SmRho1 used for immunoprecipitation experiments is especially important.

Regarding the organization of the manuscript, two of the reviewers found the combination of results and discussion confusing, and we agree that separating the extensive results from their possible implications and comparisons to other models will improve the clarity of the manuscript. The reviewers also made many other relevant editorial suggestions and identified many details that need to be clarified in the text.


We cannot make any decision about publication until we have seen the revised manuscript and your response to the reviewers' comments. Your revised manuscript is also likely to be sent to reviewers for further evaluation.

When you are ready to resubmit, please upload the following:

[1] A letter containing a detailed list of your responses to the review comments and a description of the changes you have made in the manuscript. Please note while forming your response, if your article is accepted, you may have the opportunity to make the peer review history publicly available. The record will include editor decision letters (with reviews) and your responses to reviewer comments. If eligible, we will contact you to opt in or out.

[2] Two versions of the revised manuscript: one with either highlights or tracked changes denoting where the text has been changed; the other a clean version (uploaded as the manuscript file).

Important additional instructions are given below your reviewer comments.

Please prepare and submit your revised manuscript within 60 days. If you anticipate any delay, please let us know the expected resubmission date by replying to this email. Please note that revised manuscripts received after the 60-day due date may require evaluation and peer review similar to newly submitted manuscripts.

Thank you again for your submission. We hope that our editorial process has been constructive so far, and we welcome your feedback at any time. Please don't hesitate to contact us if you have any questions or comments.

Sincerely,

Uriel Koziol
Associate Editor
PLOS Neglected Tropical Diseases

Simone Haeberlein
Deputy Editor
PLOS Neglected Tropical Diseases


Reviewer's Responses to Questions

Key Review Criteria Required for Acceptance As you describe the new analyses required for acceptance, please consider the following:

Methods
-Are the objectives of the study clearly articulated with a clear testable
hypothesis stated?
-Is the study design appropriate to address the stated objectives?
-Is the population clearly described and appropriate for the hypothesis being tested?
-Is the sample size sufficient to ensure adequate power to address the hypothesis being tested?
-Were correct statistical analysis used to support conclusions?
-Are there concerns about ethical or regulatory requirements being met?

Reviewer #1: The methods and clearly and completely described. No concerns.

Reviewer #2: -Are the objectives of the study clearly articulated with a clear testable hypothesis stated?
Not completely

-Is the study design appropriate to address the stated objectives?
Not completely (see protein purification problem)

-Is the population clearly described and appropriate for the hypothesis being tested?
-
-Is the sample size sufficient to ensure adequate power to address the hypothesis
being tested?
-
- Were correct statistical analysis used to support conclusions?
-

-Are there concerns about ethical or regulatory requirements being met?
No

Reviewer #3: The objectives are clear. Some parts of the methods are unclear, see further below.

Results
-Does the analysis presented match the analysis plan?
-Are the results clearly and completely presented?
-Are the figures (Tables, Images) of sufficient quality for clarity?

Reviewer #1: Analysis of results matches the plan. The results are clearly and completed presented. The parasite images are striking. Consider making Fig. 6 into two figures?
We feel that this figure deals with the molecular basis of the interaction between SmRho1.1 and SmHDAC8 and that dividing it into two figures is not justified.

Reviewer #2: -Does the analysis presented match the analysis plan?
Yes

-Are the results clearly and completely presented?
Not completely.
See replies to reviewer 3 and revisions carried out.

-Are the figures (Tables, Images) of sufficient quality for clarity?
Yes

Reviewer #3: Overall the results are clear, but at parts further clarification is needed.
Figure legends should be improved.
(see comments below)

--------------------

Conclusions
-Are the conclusions supported by the data presented?
-Are the limitations of analysis clearly described?
-Do the authors discuss how these data can be helpful to advance our understanding of the topic under study?
-Is public health relevance addressed?

Reviewer #1: Succinct and complete

Reviewer #2: -Are the conclusions supported by the data presented?
Not completely.
See replies to reviewer 3 and revisions carried out.

-Are the limitations of analysis clearly described?
Not completely, again, see protein purification problem.
See replies to reviewer 3 and revisions carried out.

-Do the authors discuss how these data can be helpful to advance our understanding of the topic under study?
Yes

-Is public health relevance addressed?
Yes

Reviewer #3: The conclusions are valid. Public health relevance is not addressed.
Insofar as the paper addresses the question of why SmHDAC8 is a valid and interesting target for anti-schistosomal drug development, we feel that the public health relevance of the work is established.

--------------------

Editorial and Data Presentation Modifications?
Use  this section for editorial suggestions as well as relatively minor
modifications of existing data that would enhance clarity. If the only modifications needed are minor and/or editorial, you may wish to recommend “Minor Revision” or
“Accept”.

Reviewer #1: Line 62: There are indeed 200 million individuals infected with schistosome worms, but not sure if 200 million are infected with S. mansoni.
Phrase deleted

Line 626: Typo
This part of the text has been revised.
Line706: Typo (its)
This part of the text has been moved to the Discussion and corrected.
Line 728: ‘… lysine K136 …’ seems redundant. Suggest either K136 or lysine
136. This occurs elsewhere, e.g., lines 779 – 781.
Corrected

Reviewer #2: (No Response)

Reviewer #3: Overall corrections suggested for improving the manuscript:
-        “Schistosoma mansoni” does not have to be written out every time, but rather after its first appearance, use “S. mansoni” only.
Corrected
-        Same also for “Xenopus laevis” that should be changed to “X. laevis” after its first appearance.
Corrected
-        Make sure species names are all in italics (e.g. line 163, etc)
-        Consistently use units: e.g. write mg/mL or mg mL-1 consistently (and not mg. mL-1)
Corrected throughout
-        If possible, all units of concentration should be given in molarity (especially for the treatment, do not mix mg/mL with uM)
Corrected throughout
-        Consistently use a space between number and unit (e.g. 1 mg/mL, not 1mg/mL, for example line 218)
Corrected throughout
-        Consistently use abbreviations (e.g. “aa”)
Corrected throughout
-        Consistently write “Western blot” (or “western blot”)
Corrected throughout
-        The authors should be consistent with the time forms of verbs and describe the results either all consistently in past, or in present.
Corrected throughout
-        Please indicate for every experiment, how many times it was repeated.
Corrected throughout

Further minor comments:
Abstract
-        Line 29: what is meant by “privileged target”? is it meant “potential target”?
Modified to, “has elicited considerable interest as a target..”
-        Line 30: “… Reduction of its transcription…”
Modified to, “invalidation of its transcripts..”
-        Line 32: comma after “target”
We disagree
-        Line 33/34: “… were identified in the past by…”
Modified to, “were previously identified..”
-        Line 41: introduce the term “immunoprecipitation (IP)” rather here than in line 46

-        line 55/56: “… disruption of the schistosomula…”
We have not modified the text here. We are not sure what the referee meant.

Author summary
-        line 63/64: rewrite more clearly. It should be clearly stated that resistance development is an issue, which might be induced by the MDA programs
We have added the following sentence:
“Currently one drug, praziquantel, is the treatment of choice but its use in mass treatment programs means that the development of resistance is likely and renders imperative the development of new therapeutic agents.”
```

- line 64: omit comma after “targets”

```
Done
-        line 66: replace “invalidation” by “reducing”
Modified to, “reduction in the level of transcripts of..”
-        line 70: “… novel potential anti-schistosomal drugs. The biological roles…”
Done
-        line 71: “… its protein binding partners…”
Done
-        line 73: omit comma after “here”
Done
-        line 75: omit the word “indeed”
Done


Introduction
-        line 85: To the best of my knowledge, PZQ-resistance in schistosomes is a big risk, but there is no proven resistance. Rewrite: “… and risk of resistant parasites.”
Modified to: “the emergence of PZQ-tolerant parasites and the risk of resistance.”

-        Line 94-95: omit this sentence, as the same information is given three lines further down
Done
-        Line 96: “… reduction of SmHDAC8 transcripts…”
Done
-        Reference for line 97 missing
Ref. 13 – included in text
-        Line 106: “… potential binding partners of SmHDAC8 identified, the…”
Done
-        Line 111: omit “through the binding and hydrolysis of GTP”
Done
-        Line 121: “… mutation in the budding…”
Done
-        Line 126: “… in humans…”
Done
-        Line 132: omit “have”
Done
-        Line 137: omit comma after “RNAi”
Done
-        Line 138: omit comma after “organization”
Done
Materials and Methods
-        Line 154: omit dot at end
Done
-        Line 158: “before” instead of “previously”
Done
-        Line 209: omit dot at end
Corrected
-        Line 234/244: this sentence can be omitted, as it is absolute basics
Done
-        Line 235: full stop after Coomassie Blue. Five bands…
Done
-        Line 278: rotator at which speed
A standard rotating wheel was used that turns at about 50 rpm, but we do not usually specify this.
-        Line 317: “in vitro” in italics
Done
-        Line 320: “Prof. C. Grevelding…”
Done
-        Line 337: “respectively” after “tags, “
Done
-        Line 347: source of antibodies missing
Corrected
-        Line 350-368: is there a reference available for the RNAi method applied?
Reference added: Bhardwaj R et al, Methods Mol Biol, 2011, 764:223-39
-        Line 383: “Parasites treated with corresponding amounts of Dimethyl…”
Done
-        Line 397: version of FIJI missing
Corrected

Results and Discussion
-        Line 415: instead of “They” write “SmRho1.1 and SmRho1.2”
Done
-        The sentence from line 417-420 should be moved before the sentence in line 415.
Done
-        Line 422: “CAAX motif (…”
Done
-        Line 425: check wording “anticipated”. What is meant here?
Corrected to “observed”
-        Line 433: “aa” instead of “amino acids”
Done
-        Line 440: “… metazoans, we constructed…”
Done
-        Line 442: “from vertebrates, insects, nematodes, cestodes, trematodes and ascomycetes…”
Done
-        Line 583: just “Co-IP”, no need to give here again the full term, same for “MS”
Done
-        Line 586: “… pre-immune sera…”
Corrected
-        Line 589: “… proteins of each…”
Corrected
-        Line 599: no comma before “such”
The sentence has been modified to read: “Proteins identified as SmRho1 interactors are involved in 23 different biological processes (Fig 3A), the most abundant proteins corresponding to cytoskeleton organization (19%).
-        Line 601: “… as a SmRho1 partner…”
This section has been extensively modified
-        Line 614: “… were identified in Co-IP with SmRho1”
Corrected
-        Line 618: “… SmRho1 interactome in our experiments”
Corrected
-        Line 620: Reference missing
Reference 39 has been added (Shibata et al, 2019, Small GTPases, 10:169-77)
-        Line 652: “… SmHDAC8 as SmRho1-interacting proteins”
This section has been moved to the Discussion and corrected
-        Line 656: “… masked the…”
This section has been moved to the Discussion and corrected
-        Line 659: “… to be kept in mind…”
This section has been moved to the Discussion and corrected
-        Line 660: delete “in fact”
This section has been moved to the Discussion and modified
-        Line 661: Reference missing
This section has been moved to the Discussion and ref. 11 added.
-        Line 663: “in vitro” in italics
This section has been moved to the Discussion and corrected
-        Line 667: “… all the known protein partners of human RhoA…”
Corrected
-        Line 754/755: this sentence has to be rewritten. It is not clear, what the authors want to state here.
This section has been rewritten and moved to the Discussion. However, we have decided to remove the sentence. We agree that we cannot conclude that only one isoform is acetylated.
-        Line 769: “… and showed…”
Done
-        Line 786: “… SmRho1.2 were incapable…”
Corrected
-        Line 845: “… specifically to SmDia”
We disagree; proteins interact with each other, not to each other.
-        Line 846: “As we have shown a specific…”
Again, “because” is much maligned and in consequence underused.
-        Line 848: “… and the Rho Binding…”
Corrected
-        Line 849: “… using the Y2H…”
Corrected
-        Line 850: “In Fig7A we show…”
Corrected
-        Line 859: “… requires further investigation.”
Corrected


Conclusion
The Conclusion has been integrated into the new Discussion section.
-        Line 1064/1065: “… and their partners, it should be kept in mind…”
We disagree with the correction. “Borne in mind” combines the idea of “carry” with “hold” or “keep”.
-        Line 1069: “… allow to obtain…”
We disagree with the correction. We have substituted (now in the Discussion section, Line 1175): “would allow obtaining conditional..”


Figure 1
-        Legend very well and clearly written
-        Remove lines around subfigures
Done
-        Line 513: “… X = terminal AA)”
Corrected
-        Line 516: “human”
Corrected
-        Line 518: “semi-conserved”
Corrected
-        Line 521: “72% and 73% respectively”
Corrected
-        Line 525: could the 310-helices be shown in another color than cyan to distinguish better from alpha-helices?
Changed to green
-        Line 691: only “Co-IP” not full wording needed
Corrected
-        Line 733: “… expression systems (Fig. 6).”
Corrected
-        Line 734: “right panel”
Corrected
-        Line 735: “… SmRho 1.1 could grow…”
Corrected
-        Line 740: “… confirm the specific…”
Corrected
-        Line 742: “… it will be necessary…”
Corrected
-        Line 745: “MS” instead of “mass spectrometry”
Corrected
-        Figure 10 is stated first in line 866, before Figure 8. Thus the numbering of the figures should be chronologically adapted.
This section has been moved to the new Discussion section, solving the problem.
-        Line 902: “… of the parasite…”
Corrected
-        Line 911: “… We could, however, observe that TH65 seemed to…”
Corrected
-        Line 915: “… TSA were highly…”
Corrected
-        Line 918: “… revealed…”
Corrected
-        Line 923: “… on the actin cytoskeleton…”
Corrected
-        Line 924/925: “… that the effects of TH65 could be due to…”
Corrected
-        Line 931: “… organization, but in schistosomula we observed…”
Corrected
-        Line 938: comma after “enzyme”
Corrected
-        Line 939: “… we can nevertheless not…”
Corrected
-        Line 945: “… we will also…”
Corrected

Figure 2:
-        As the figure contains information on Rho1 of many different species, it would be helpful for the reader, if you would highlight the Schistosome proteins (for example with *).
Corrected
-        Line 578: “in red rectangle”
Corrected


Figure 3:
-        It would be preferred, if the text was bigger, and the pie chart smaller
The Figure has been modified.
-        In the legend, the information on the % given is missing
Corrected

Figure 4:
-        Line 716/717: “… were immunoprecipitated respectively…”
Corrected
-        Line 720: state which IgG mouse antibody was used for the MOCK experiment (also not given in the Methods section)
Corrected
-        Legend is incomplete. How much protein was loaded? Clearly indicate, which ones are the eluates.
The legend has been improved to specify the eluates and input lanes. Numbers of parasites used are indicated in the Materials and Methods and the amount of IP eluate loaded is now indicated in the legend
-        How many times were the tests repeated? Please indicate.
The figure shows the results of one experiment representative of three carried out. This is now specified.

Figure 5:
-        line 729: “… spectrum of SmRho1…”
The legend has been modified and corrected
-        legend incomplete for this figure. What do the colors mean?
The significance of the colors is now specified in the legend
-        How many times were the tests repeated? Please indicate.
The experiment was performed twice on adult worms obtained by three separate perfusions of infected hamsters. This is specified in the legend.

Figure 6:
-        legend incomplete. Methods and results are repeated here, but no clear description of the figure is given.
The legend has been revised
-        Subfigure a: line 818(819: “… medium (left panel) and diploids… medium (right panel).”
Corrected
-        In subfigure B, indicate “SmHDAC8-Myc” and “SmRho1-HA”, instead of only “Myc” and “HA”
Corrected
-        Subfigure C: mutants do not appear in same order in figure and legend, please correct
The order of samples in the blot cannot be changed and if the order of the mutants in subfig. C is changed, the comprehension of how they were synthesized will be adversely affected. To help with this, arrows have been added to subfig. C
-        Subfigure C: line 835: “given in red…”
Added to the legend.
-        Why is this figure given in two parts with two titles? Maybe the figure should then rather be given as two separate figures, if sub-titles are needed
The overall title has been modified and the subtitle removed.
-        How many times were the tests repeated? Please indicate.
Now specified in the text: “Experiments were performed three times on oocytes from three different females.”

Figure 7:
-        legend incomplete. Methods and results are repeated here, but no clear description of the figure is given.
The legend has been modified.
-        Subfigure B, line 890: “Co-IPA”
Corrected
-        In subfigure B, indicate “SmHDAC8-Myc” and “SmRho1-HA”, instead of only “Myc” and “HA”
SmHDAC-Myc does not appear in the figure. We have specified, “SmDia-RBD (Myc tagged) and SmRho1.2 (HA-tagged) in the legend.
-        How many times were the tests repeated? Please indicate.
Now specified in the text: “Experiments were performed three times on oocytes from three different females.”


Figure 8/9:
-        Indicate the concentrations of inhibitors also in the Figure with the respective inhibitors. E.g. “TH65 (50 uM)”
Done
-        It is unclear, which negative control was used for what (DMSO/water)
Both were used as negative controls for the inhibitors.
-        Give all the inhbitors in uM.
Done
-        Clearly state the names of the RNAi sondes also in the figure, as stated in the figure (dsLuc, dsSMHDAC8, dsSmRho1)
Corrected
-        “… Actin-F was stained with phalloidin (green)”… “DAPI (blue)”.
Corrected
-        How many times were the stainings performed?
In each case experiments were performed three times on adult worms obtained by three different hepatic portal perfusions of hamsters. This information has been added in the text
 How many times was the qPCR performed? Please indicate. Also, if means and SDs are given in subfigure B.
Corrected, now specified in the legend.

-        Line 1036: “… bar represents…”
Corrected
Figure 10
-        Very nice overview, but the figure is very incomplete. Much more information on the figure should be given here.
We have modified and simplified the figure and included a much fuller description in the text and the legend.
-        Line 1084: comma after “SmRho1.1”
Corrected

--------------------

Summary and General Comments
Use  this section to provide overall comments, discuss strengths/weaknesses of the study, novelty, significance, general execution and scholarship. You may also include additional comments for the author, including concerns about dual publication, research ethics, or publication ethics. If requesting major revision,please articulate the new experiments that are needed.

Reviewer #1: The manuscript by Pierce and co-workers examines the mechanism of killing after inhibition of HDAC 8 in Schistosoma mansoni. HDAC8 is a validated and druggable target for schistosomiasis drug development. The study is timely, important, well presented, and well written. Linking the activity of HDAC inhibition to small GTPase Rho1 are clear and compelling. The manuscript is very long and presents a very large amount of data; it might be easier to handle as two stories, but the studies are complimentary and could be published as is.
Disruption of actin was more apparent in schistosomula than adults, but HDAC inhibitors are active against both worm stages. How is this interpreted?
We agree that we should have dealt with this issue and we have added a paragraph on this point in the new Discussion section.

Reviewer #2: The paper of Pagliazzo et al. deals with the further characterization of Histone deacetylase 8 (SmHDAC8) and its potential interaction partners in S. mansoni. This an important topic of schistosome research in the context of basic biology but also towards the identification of potential targets and the development of new drugs.
The Pierce group has much expertise in this field, and here they describe the molecular and cellular characterization of two isoforms of SmRho1, SmRho1.1 and SmRho1.2 as potential SmHDAC8 partners. Among others, the authors found evidence for a role of SmHDAC8 in cytoskeleton organization, a process in which SmRho1.1 may be involved.
This is an interesting study with a number of novel results that will be of interest to readers of PLoS NTD. Furthermore, the results extent existing knowledge about Rho signaling-pathways in schistosomes. However, before the manuscript can be considered for publication the authors must address the following major and minor points.
Major comments:
Lines 192 ff: protein purification worked to some extent. However, the purified protein appears not to be “clean”; there are additional proteins co-purified that have been subjected to mice as well for antibody production. Here the statement of “monospecificity” (line 213) seems not to be justified.
The authors should comment on this, and they should discuss in the paper whether this impurity might have influenced their results such as the Co-IP experiments and the subsequent MS analysis.

This is a very valid point and we were clearly wrong to use the term “monospecificity”. We have modified this and added comments in the methods section. We have also been more careful to qualify our comments in discussion of the interactome. The WB after affinity purification of recombinant SmRho1.1 shows that the protein was not completely pure (Fig; S2), raising the possibility that contaminating proteins may have led to the production of antibodies cross-reacting to schistosome proteins other than SmRho1. However, we have performed an experiment in which recombinant SmRho1.1 was used to pre-absorb anti-SmRho1 antibodies. The results (included in supplementary Fig.2D showed that the major 22 kDa band was depleted whilst the extraneous bands were unaffected. This suggests that the latter are produced by cross-reacting antibodies present in the mouse serum.  It is possible that the antibodies directed against SmRho1 could also recognize other SmRho family proteins, notably SmRho2, but these were not always detectable and were not visible on the blot of the depletion experiment. We have commented on these issues in the text. Nevertheless, the MS analysis of the CoIP experiments and the depletion experiment clearly show that the antibodies recognized SmRho1 and we should point out that both isoforms were recognized.  Therefore, we feel that our results concerning the potential protein partners of SmRho1 are valid, particularly in respect to cytoskeleton elements.


Line 606 ff: the authors argue about a parasite protein complex consisting of SmRho1, SmROCK1, SmLIMK, and the Myosin light chain. If it exists, one would expect that these partners share the same compartments/organs for expression. Have the authors searched in the SchistoCyte Atlas (http://www.collinslab.org/schistocyte/) and/or other sub-transcriptome resources to find additional support for their assumption? If not, this has to be done, and the results reported.

As suggested by the reviewer we have searched the SchistoCyte Atlas. However, we found no relevant data that allow us to link these proteins in the parasite. We are open to further suggestions, but for this revision we have not included our analysis.

Generally, the results are filled with hints to others studies when molecules and their potential functions are reported. Much of this is discussion/conclusion content and should be remove from the results section, which should focus on the obtained data.

We have created a new Discussion section and removed the discussion elements from the Results. We agree that this increases clarity.

 Furthermore, there are many “ifs and buts” in the results section, which should be removed or rephrased to avoid losing trust and credibility in this study.

We have revised the Discussion as stated above and have been as definite in our conclusions as the Results allow. However, some of our conclusions are necessarily speculative and we cannot remove all the “ifs and buts”.

This also applies to the conclusion in lines 665-666, in which the authors claim that their (so far presented) results show SmRho1 involvement in cytosolic processes regulating cytoskeleton function, as does the human ortholog, HsRhoA. This conclusion requires deeper analysis that is much more functional, and it is not justified based on the interaction partner analysis, not only because the analysis was based on partners identified by Co-IP with an antibody whose purity was low. The authors should tune down their functional claims here and elsewhere, as they do not have sufficient experimental support for this. Supporting evidence for SmHDAC8-
SmRho1.1 interaction (and SmRho1.2-SmDia, a different story) is presented later, and it is restricted to these tandem combinations.

The involvement of SmRho1/SmHDAC8 in cytoskeleton organization is not based only on interactome analysis but also on the observed effects of inhibitors and transcript knockdown. We agree that the antibody was not monospecific (see previous reply) but disagree that the purity of the recombinant protein used of its generation was “low”. We have been careful to limit our claims about function, whilst avoiding “ifs and buts”, in the revised Discussion.

Line 905: TSA inhibits class I and II mammalian histone deacetylases. How many of these deacetylases (paralogs) exist in S. mansoni? The authors should comment in their manuscript about additional potential inhibitor targets in S. mansoni. In case more than one HDAC paralog exist, have the authors checked and confirmed that the dsRNA part used is specific for their gene?

We have previously reported the repertoire of class I and II (and III - sirtuins) HDACs in schistosomes (ref 11 for the class I enzymes) and did not feel it necessary to reiterate this information, apart from the class I enzymes that we mention in the Introduction. SmHDAC8 is very dissimilar to the more closely related SmHDAC1 and 3 and there are no close paralogues encoded in the S. mansoni genome. Cross-reactivity of RNAi between SmHDAC8 and the other two class I enzymes is not possible and the same is the case for the class II HDACs.


And how do these phenotypes compare to other studies using HDAC8 inhibitors in S. mansoni, such as the one from Saccocia et al. 2020 (PMID: 31661956), which the authors fail to cite?

It is difficult to compare our work with that of Saccoccia et al since the latter concentrated their analysis of phenotypic effects of their inhibitors on the reproductive apparatus of the parasites using carmine red staining and were not concerned with the cytoskeleton. This is why we did not cite their article.

Line 935-935: With 22%, knock-down efficiency in adults, silencing of SmHDAC8 cannot be considered as robust. How efficient was silencing efficiency of rho1 in somules and adults, and did dsRNA discriminate between the isoforms?

The level of knockdown in adult worms was much higher; the figure of 22% refers to the schistosomula. We have discussed this conundrum in our reply to Referee 1 and have commented on it in the Discussion. The dsRNA used did not discriminate between the two isoforms, but we were able to analyze the degree of knockdown achieved separately by q-RTPCR.

Fig. 10: The authors mentioned a previous study about a proven SmDia-SmTK3 (Src-likeTK) interaction. How does this fit into the scenario?

In the Discussion we mention that in humans the RhoA-GTP/mDia/Src complex participates in the regulation of actin filaments, suggesting that the same may occur in schistosomes, but we cannot speculate further on this.


Minor comments
Lines 163/325 elsewhere: species names should be given in italics; this applies also to part of the names of restriction enzymes, which originate from species. E.g., in HindIII the first three letters should be in italics. Please check the whole manuscript and correct where needed.
Corrected

Lines 170-following: which source was used for primer design before RACE was planned, WormBase ParaSite or something else? And which genome version was used, V5 or V7?

Our primers were generated using the sequences of the partial cDNA clones obtained after Y2H screening. This has been specified in the Methods section. Primer design for RACE-PCR was ad hoc.

 Lines 350 ff.: no reference is given for this kind of RNAi approach (one of several); please cite original literature.

We have added a reference to the Methods in Molecular Biology paper by Bhardwaj et al, 2011.

Line 378: a Smp identifier should be added to the used reference gene. The citation is not appropriate, the cited paper seems not to be from the schistosome field. Please cite the paper, in which it has been demonstrated that PSMB7 is a suitable reference gene for S. mansoni.

Smp identifier has been added and the reference corrected (now ref. 32, Haberlein et al 2019).

Lines 381/383: … 4/2 microgram or 4/2 micromolar? Please, harmonize units.
Corrected

Line 411: provide the Smp identifiers for Rho1.1 and 1.2.
We have added the Smp identifier for SmRho1.1. For SmRho1.2 no identifier exists yet.

Line 448: to support their assumption of the phylogenetic analysis, the authors should include the orthologs of S. rodhaini, the closest relative of S. mansoni (see Lawton et al. 2011, PMID: 21736723).
Unfortunately, most of the Rho sequences available for S. Rhodaini are poorly annotated or incomplete. We have therefore preferred not to include them in our (already extensive) tree.


Line 601: add Smp identifier for cofilin (and also for all other S. mansoni
gene/proteins reported about).
We have included NCBI accession numbers and Smp numbers for all these proteins

Line 612: “… once again”? May be I missed it, but where have the authors
illustrated the involvement of the GTPase in the regulation of cytoskeletal events before?

The text has been extensively revised in this part of the manuscript.

Line 661: is there a reference for the statement of low SmHDAC8 expression in adults?
The reference has been added – it is ref 11.

Lines 911-912: correct grammar “We can … observed” … .
Better: We have observed, or simply: TH65 seemed to impact …
Corrected

References: missing italics with species names
Corrected
Reviewer #3: Pagliazzo and colleagues here report on an impressive study on the characterization of the Schistosoma mansoni protein SmHDAC8 and in particular its interaction partners, mainly SmRho1. They hereby build on a previous study, where interaction of SmHDAC8 and SmRho1 was already identified. The authors here performed further analyses of the SmRho1 isoforms SmRho1.1 and SmRho1.2 on adult schistosomes and schistosomulae and show by Y2H experiments, and expression in Xenopus oocytes, that SmRho1.1 (not SmRho1.2) directly interacts with SmHDAC8. They follow-up on this
with several SmRho1 mutants and find that the C-terminal part of SmRho1.1 is important for the interaction. Further, the authors show via Y2H mating experiments, and expression in Xenopus oocytes that SmRho1.2 interacts with SmDia. By immunofluorescence of parasites where SmHDAC8 or Rho1 were inhibited or knocked-down, they show that SMHDAC8 and SmRho1 is involved in cytoskeletal organization, in particular in schistosomulae.

Overall, the study is quite well written and follows a clear structure. The authors chose to merge results and discussion, which makes it difficult, at parts, to understand what was clearly performed in the present study, and what is rather discussion/speculation or found in other studies. Even though a merge of results and discussion is generally accepted in the PlosNTD guidelines, for the present study, a clear separation of results and discussion would be preferable.

We agree and have separated the Results and Discussion sections to improve clarity


Most parts of the study are clear, but at parts, further clarification is needed.
We have made a major effort to improve the manuscript in this respect, based on reviewers’ comments

Some suggestions for improvement of the language, to make things more clear, are given further below (minor points). Most of the references are given, but some are missing, as also indicated below.

Major points to be clarified
-        The abstract does not include all the major points addressed in the study and should be improved.

We have slightly revised the Abstract within the limits of the allowed word count.

-        For the Method section, it should be stated what is the name of the here applied S. mansoni isolate (line 155).
The NMRI strain is used in the lab (and has been for about 60 years! This has been specified

-        In line 169 it is missing, for how long the oocytes were kept at 19°C.
The oocytes were kept at 19°C for 2 hours. This has been added.


-        Is there a reference available for the provided plasmid from Marek and Romier (line 196)?
The reference has been added (ref 13)


-        The section from line 226-229 is unclear, as the concentration of the antibody is not given, or at least the ratio of how it was diluted in beads should be stated.

We have specified the volume of serum and the quantity of beads (5 mg) used.

-        In the section from line 300-308 the authors should check again the mutant description, it seems that EMNN and QVKS were mixed up.
We have checked and there does not seem to be a mistake. Note that the CoIP experiments with these two mutants are described in Fig.S5.

-        The authors note in line 592 that it might be that protein quantities were differing between experiments. What were the protein amounts used for each experiment? Were internal control proteins used for normalization?
In CoIP experiments no internal controls were used. In general marked differences in peptide frequencies are expected. Moreover, the differences seen between the two experiments show that other factors than protein amounts determine the results obtained.

-        In line 595 the authors write about 86 and 32 proteins that were retrieved in two separate IPs. The authors should (at least in a supplementary table) show this data, and also show, how many and which of the proteins were common in the two setups (e.g. in a Venn diagramme).

A Venn diagram has been added to Fig. 3. The data on the proteins identified by CoIP/MS is contained in the S3 Table.

 Following this, in line 599, it is unclear, about which protein set the authors are writing when it is stated “These proteins are involved
in…”. Are these all the proteins from the two IPs, or only the shared ones?
Thus, this part of the results section needs further clarification.

 This refers to proteins identified in either of the experiments and has been clarified in the text.

-        The authors backed up their MS data with Western blots with SmHDAC8 and SmRho1 (Fig 4). As they state, SmROCK they did not detect as an interacting protein in their MS data, though expected. Did the authors check the presence of SmROCK in the interactome of SmRho1 by Western blot?

Unfortunately this was impossible since we lacked antibodies to SmROCK. We did attempt to clone SmROCK, but this proved intractable.

-        The authors checked the acetylation of SmRho1 by MS. Why was that not done for SmRho1.1 and SmRho1.2 separately?

The acetylated lysine is shared by both isoforms and the antibody used for CoIP recognizes both. We could not identify specific peptides for each isoform in the analysis.

-        The figure legends are clear in the beginning, but from figure 4 on, the legends appear incomplete.

Figure legends have been extensively revised according to the comments of Reviewer 2

-        From line 739 on, the authors discuss that the specificity of interaction between SmHDAC8 and SmRho1.1 would have to be further confirmed by IP. Why was this not included in this study?

CoIP on schistosome extracts followed by MS analysis to determine the SmRho1 isoform interacting with SmHDAC8 proved impossible to accomplish in the time available to us. It would represent a significant investment that we cannot currently envisage.

-        Why was the efficacy of RNAi so low for schistosomulae (Figure 9)?

Previous results in our group and from others has shown that the soaking method used for RNAi in schistosomula gives varying results. In this case the results were mediocre. Nevertheless, even a relatively low level of knockdown can produce a significant biological effect. See for example Mourao MM et al., PLoS Negl Trop Dis, 2009, 3(8):e502

-        Why were different concentrations of inhibitors used for adult worms and schistosomulae (Figures 8/9)?

Concentrations were based on preliminary studies (not shown in the manuscript) aimed at determining doses and times of exposure that allowed us to observe effects on the actin cytoskeleton without accompanying induction of necrosis/apoptosis. These were different for the two life-cycle stages.


--------------------
Additional comments from editors to be addressed:

- Figure 4A - It seems likely that in the left panel, the labels "IP SmHDAC8" and "IP SmRho1" were incorrectly exchanged
No, there is no error

- Figure S3 and S4 - the legends are exchanged as well

 The legend to S4 has been corrected. The legend to S3 is correct.

- Figures do not appear in order in the main text (e.g. Fig. 10 appears before Fig. 8).

This has been corrected. The first reference to Fig. 10 now appears in the Discussion.

- Regarding the interaction of SmRho1.2 and SmDia, how do you interpret this result considering that Quack et al 2009 found an interaction by Y2H using (presumably) SmRho1.1, although in mutant activated versions?

The version of SmRho1 used by Quack et al is not exactly identical to either SmRho1.1 or SmRho1.2. We are not sure what the Quack et al transcript corresponds to and, given this doubt, we felt that we could not comment.

- Regarding figure 8, the main text mentions the actin cytoskeleton of the tegument, but the figures also show the actin staining of the subtegumental muscle fibers.

See comment below

- Regarding figure 9, it is clear that the muscle cells are affected by the
treatments - however, at this level of magnification, it is not easy to say what happens regarding the structure of actin filaments (l.920), and the gaps in staining could be for example due to death or deattachment of the muscle cells.

We agree and we have added a comment in the Discussion on this point.

- Regarding figure 9, the orthogonal views presented in B could be included with the projections shown in A.

A modified version of the figure has now been included.

--------------------

PLOS authors have the option to publish the peer review history of their article (href="https://journals.plos.org/plosntds/s/editorial-and-peer-review-process#loc-peer-review-history"
target="_blank">what does this mean?. If published, this will include your full peer review and any attached files.

If you choose “no”, your identity will remain anonymous but your review may
still be made public.

Do you want your identity to be public for this peer review? For information about this choice, including consent withdrawal, please see our <a href="https://www.plos.org/privacy-policy" target="_blank">Privacy Policy</a>.

Reviewer #1: No

Reviewer #2: No
Reviewer #3: No


Figure Files:
While revising your submission, please upload your figure files to the Preflight Analysis and Conversion Engine (PACE) digital diagnostic tool,
https://pacev2.apexcovantage.com. PACE helps ensure that figures meet PLOS
requirements. To use PACE, you must first register as a user. Then, login and navigate to the UPLOAD tab, where you will find detailed instructions on how to use the tool. If you encounter any issues or have any questions when using PACE, please email us at figures@plos.org.

Data Requirements:
Please note that, as a condition of publication, PLOS' data policy requires that you make available all data used to draw the conclusions outlined in your manuscript.
Data must be deposited in an appropriate repository, included within the body of the manuscript, or uploaded as supporting information. This includes all numerical values that were used to generate graphs, histograms etc.. For an example see here:
http://www.plosbiology.org/article/info%3Adoi%2F10.1371%2Fjournal.pbio.1001908#s5.

Reproducibility:
To enhance the reproducibility of your results, we recommend that you deposit your laboratory protocols in protocols.io, where a protocol can be assigned its own identifier (DOI) such that it can be cited independently in the future.
Additionally, PLOS ONE offers an option to publish peer-reviewed clinical study
protocols. Read more information on sharing protocols at
https://plos.org/protocols?utm_medium=editorial-email&utm_source=authorletters&utm_campaign=protocols

__________________________________________________
In compliance with data protection regulations, you may request that we remove your personal registration details at any time.  (Use the following URL:
https://www.editorialmanager.com/pntd/login.asp?a=r). Please contact the publication
office if you have any questions.
```
